# Attenuated Chimeric GI/GIII Vaccine Candidate against Japanese Encephalitis Virus

**DOI:** 10.3390/vaccines11121827

**Published:** 2023-12-08

**Authors:** Eunji Lee, Minjee Kim, Young Bong Kim

**Affiliations:** Department of Biomedical Science and Engineering, Konkuk University, Seoul 05029, Republic of Korea; eunjj123@naver.com (E.L.); mj0411@konkuk.ac.kr (M.K.)

**Keywords:** Japanese encephalitis virus, vaccination, reverse genetics system

## Abstract

Japanese encephalitis (JE) is a very severe disease characterized by high fatality rates and the development of permanent behavioral, psychiatric, and neurological sequelae among survivors. Japanese encephalitis virus (JEV), a flavivirus, is responsible for JE. In Asia, Genotype I (GI) has emerged as the dominant strain, replacing Genotype III (GIII). However, no clinically approved drug is available to treat JEV infection, and currently available commercial vaccines derived from JEV GIII strains provide only partial protection against GI. Utilizing a reverse genetics system, this study attempted to produce a novel chimeric JEV strain with high efficacy against JEV GI. Accordingly, a GI/GIII intertypic recombinant strain, namely SA14-GI env, was generated by substituting the E region of the GIII SA14-14-2 strain with that of the GI strain, K05GS. The neurovirulence of the mutant virus was significantly reduced in mice. Analysis of the immunogenicity of the chimeric virus revealed that it induced neutralizing antibodies against JEV GI in mice, and the protective efficacy of SA14-GI env was higher than that of SA14-14-2. These findings suggest that SA14-GI env may be a safe and effective live-attenuated vaccine candidate against JEV GI.

## 1. Introduction

The Japanese encephalitis virus (JEV) is a spherical, positive-sense, single-stranded, non-segmented RNA virus (Flaviviridae family) that encompasses other viruses, such as tick-borne encephalitis, West Nile, dengue, and yellow fever viruses [1,2]. JEV was first detected in Japan in 1924, followed by several other Southeast Asian countries, and is currently the leading cause of epidemic encephalitis [3]. Epidemic encephalitis is a re-emerging zoonotic infectious disease that involves a wide range of activities and has spread to Russia, Africa, and Australia [4,5]. The mosquitoes of the genus Culex serve as carriers for JEV. Approximately 68,000 people worldwide are infected annually by JEV, but most remain asymptomatic [5]. However, among patients manifesting symptoms, approximately 30% die, and 30–50% of survivors continue to have neurological complications [6]. The virus incubation for the infection is between 7 and 14 days [6,7]. Unlike in adults, who are asymptomatic upon infection, in children and the elderly, Japanese encephalitis (JE) causes high fever, headache, perceptual abnormalities, disturbance of consciousness, coma, and death. In addition, during the recovery period, patients may experience sequelae, such as speech impairment and impaired judgment [8]. The virus is transmitted from mosquito to human through the bite of infected mosquitoes. However, humans are incidental and dead-end hosts. The virus persists in the body for only a few days and with low titers; hence, there is no possibility of infection through transfusion from patient–mosquito bites or person-to-person transmission [8].

The approximately 11 kb genome of JEV is initially translated as a singular polyprotein, which is subsequently cleaved by both host and viral proteases to generate three structural proteins, such as capsid (C), premembrane (prM), and envelope (E), along with seven non-structural proteins (NS1, NS2A, NS2B, NS3, NS4A, NS4B, and NS5), including the enzymes and factors required for RNA genome replication [9]. Similar to other flaviviruses, the genomic RNA of the JEV is packaged by C protein, forming a nucleocapsid. This nucleocapsid is enveloped by a lipid bilayer derived from the host cell containing E and prM [10].

The JEV is categorized into five genotypes (GI–GV) based on the nucleotide database of the C/PrM and E proteins [11]. From the time it first emerged in Japan in 1924 until the late 20th century, Genotype III was the dominant circulating genotype associated with human infections. However, Genotype I has since become the dominant strain. All of the available JEV vaccines are based on the GIII strain [11]. There are four types of JEV vaccines: inactivated mouse brain-derived JEV, Vero cell-produced JEV, live-attenuated JEV, and live-attenuated chimeric YFV-17D/JEV vaccines [11]. However, these vaccines are based on the GIII strain that provides partial protection against JEV GI [12]. Because of the prevalence of GI strains, the development of novel JEV vaccines must be expedited to manage and contain this epidemic effectively.

Reverse genetics is a genetic manipulation that involves cloning replicable viral RNA or DNA to create a complete artificial viral genome, which is then introduced into a cell to produce new viruses. The reverse genetics system involves several processes during which the infectious cDNA corresponding to the RNA genome of the JEV is synthesized and engineered into a specific plasmid [13]. RNA viruses have rapid replication rates and are highly adaptable, enabling them to survive in various environments. RNA polymerase, which synthesizes RNA from a DNA or an RNA template, does not have proofreading activity during transcription, resulting in high errors at a rate of 1 per 1000 to 100,000 nucleotides [14]. In other words, RNA polymerase is more error-prone than DNA polymerase, which makes it difficult to develop a vaccine because the virus is prone to mutating as it replicates. Therefore, a platform that can analyze these viruses rapidly and develop effective antiviral drugs and vaccines is necessary. Subsequently, in vitro transcription is employed to produce a full-length viral RNA transcript and transfected into cells to produce infectious recombinant JEV virions [13,15].

Here, we adopted a reverse genetics system (GIII to GI) to construct infectious cDNA clones and produce chimeric JEV. We also analyzed the characteristics and immunogenicity of the viruses through the animal study and attempted to develop a vaccine that may produce a protective effect against JEV.

## 2. Materials and Methods

### 2.1. Cell Culture

Vero cells, which originated from the African green monkey kidney, were obtained from the American Type Culture Collection (ATCC; CCL81, Manassas, VA, USA). The Vero cells were cultured at 37 °C in 5% CO_2_ in Dulbecco’s modified Eagle’s Medium (DMEM) in 10% fetal bovine serum (FBS) and 1% penicillin/streptomycin (Gibco, Grand Island, NY, USA). The baby hamster kidney fibroblast cell (BHK-21) lines stably expressing T7 RNA polymerase (BHK-T7) were maintained in DMEM under the abovementioned conditions.

### 2.2. Viruses

Two strains, the GI JEV strain K05GS and SA14-14-2, were provided by the Centers for Disease Control and Prevention of Korea. The GI JEV strain K05GS (GenBank accession no. KR908702) was isolated in Korea in 2005. The SA14-14-2 is a live-attenuated JEV vaccine strain (GenBank accession no. JN604986) that was originally derived from the wild-type strain SA-14, which belongs to Genotype GIII. The virus was amplified and harvested in Vero cells when the cytopathic effect (CPE) was approximately 70–80% after three rounds of freeze-thawing.

### 2.3. Construction of Chimeric Virus

The JEV GI/GIII intertypic virus was constructed with the K05GS envelope protein in the SA14-14-2 backbone. The prM/E proteins of the K05GS strain were codon-optimized and cloned into the pBHA vector with Bioneer to effectively express the target genes in humans. The cDNA of JEV SA14-14-2 was copied into the pACYC184 vector, and the E region of the infectious cDNA clone SA14-14-2 was changed with the in vitro-synthesized E region of Syn_GI. The plasmid that the prM/E region of the K05GS strain codon-optimized was Syn_GI, and it was subcloned into the pACYC184 vector by Bioneer. The E regions of K05GS and SA14-14-2 backbones were amplified with specific primers (Table S1), and each DNA fragment was cloned into the corresponding region with the In-Fusion Cloning System (Takara Bio, Seoul, Korea). After the cloning, plasmids were amplified from *Escherichia coli* HST08 cells.

To create the virus, we conducted transfection in BHK-T7 cells with Lipofectamine^®^ 2000 Reagent (Invitrogen™, Carlsbad, CA, USA). Cells were maintained in DMEM with 10% heat-inactivated FBS before transfection. After three days, the transfected cells were thawed thrice to obtain the viral supernatants. The complete recombinant infectious clone was termed SA14-GI env.

### 2.4. Plaque Assay

Vero cells were seeded (2 × 10^5^ cells/well) in six-well plates. The cells were maintained at 37 °C with a 5% CO_2_ chamber for 24 h. The serially diluted viruses were inoculated into Vero cells for 1 h under the same atmospheric environment. The inoculum was discarded, and a DMEM-based overlay medium containing 2% low-melting agarose (SeaPlaque™ Agarose, Lonza, Rockland, ME, USA) and 2% FBS were added to the wells. The cells were stained with a 1% crystal violet solution after 6–7 days of incubation, and the viral titers were calculated in plaque-forming units (PFUs)·mL^−1^.

### 2.5. Mouse Challenge Experiment

The animal experiments were performed at the BL2 facility after approval by the Institutional Animal Care and Use Committee (IACUC Approval No.: KU23105) of Konkuk University. Female BALB/c mice were purchased from Orient-Bio (Seungnam, Korea) and utilized for challenge tests. The neurovirulence analysis mice groups (4 weeks old, n = 6) were injected intracerebrally (i.c.) with 40 μL (1 × 10^4^ PFU) of virus (K05GS, SA14-GI env, and SA14-14-2) diluted in phosphate-buffered saline (PBS). Survival rates and body weights of the mice were monitored for 14 days.

For immunogenicity analysis, BALB/c mice were utilized to validate the vaccine candidate’s protective efficacy, and K05GS, a JEV strain belonging to GI, was utilized for the virus challenge. Three mice groups (6 weeks old, n = 6) were immunized twice, intraperitoneally (i.p), with 200 μL (1 × 10^5^ PFU) of each virus strain at 2-week intervals. One week after each vaccination, the mice’s blood samples were collected, and the obtained serum was utilized for serum antibody levels. Mice were i.c. challenged with 100 LD_50_ of GI K05GS two weeks after the last vaccination and monitored daily for 14 days.

### 2.6. Enzyme-Linked Immunosorbent Assay (ELISA)

The JEV-specific antibody levels in immunized mice were measured with an enzyme-linked immunosorbent assay (ELISA). A 96-well immuno-plate (Thermo Fisher, Waltham, MA, USA) was coated with 50 μL (1 × 10^3^ PFU) of the inactivated K05GS strain of JEV in coating buffer (0.05 M sodium carbonate, pH 9.6) and incubated at 4 °C. The plate was blocked with 5% skim milk for 1 h. After the blocking, the mouse serum was added and incubated at room temperature for 3 h. The horseradish peroxidase (HRP)-conjugated polyclonal goat anti-mouse IgG (Abcam, Cambridge, UK) was diluted to 1:10,000 and added to each well. Between each step, it was washed with PBS-T (0.05% Tween 20 in PBS) thrice. After the conjugation step, the TMB solution (Invitrogen™, Carlsbad, CA, USA) was added, and the reaction was stopped with 1N H_2_SO_4_. Finally, the absorbance was evaluated with a microplate spectrophotometer (Bio-Tek Instruments, Winooski, VT, USA) at 450 nm.

### 2.7. Micro-Neutralization Assay

The assays were conducted with Vero cells with serum heat inactivated at 56 °C for 30 min. Vero cells were seeded at a density of 1 × 10^4^ cells in a 96-well plate environment before infection. A total of 100 TCID50 JEV strains (K05GS) were mixed at a 1:1 ratio with two-fold serial dilutions (1:10 to 1:640) of sera from mice immunized with each viral strain and incubated at 37 °C for 1 h. Then, the mixture was added to Vero cells cultured in 96-well plates for 1 h. After absorption, the culture medium containing 2% FBS was added and incubated for 6–7 days. After the incubation, the cells were stained with 1% crystal violet, and the neutralization antibody titers (NT50) were defined as the dilution factor corresponding to 50% of the wells not exhibiting CPE relative to the control utilizing a microplate spectrophotometer at a wavelength of 450 nm.

### 2.8. Statistical Analysis

All statistical analyses were conducted with GraphPad Prism 8.0.2 (GraphPad Software, Inc., San Diego, CA, USA). All the experimental data were displayed as mean ± standard deviation (SD). The group comparison data were analyzed with one-way ANOVA and the Tukey–Kramer post hoc test (*p* < 0.05 considered significant).

## 3. Results

### 3.1. Construction of Infectious, Full-Length cDNA Clone of Chimeric JEV

A chimeric plasmid (JEV) was constructed from genes encoding structural/non-structural proteins downstream of the T7 promoter, allowing it to transcribe and transfect the BHK-T7 cells.

**Figure 1:**
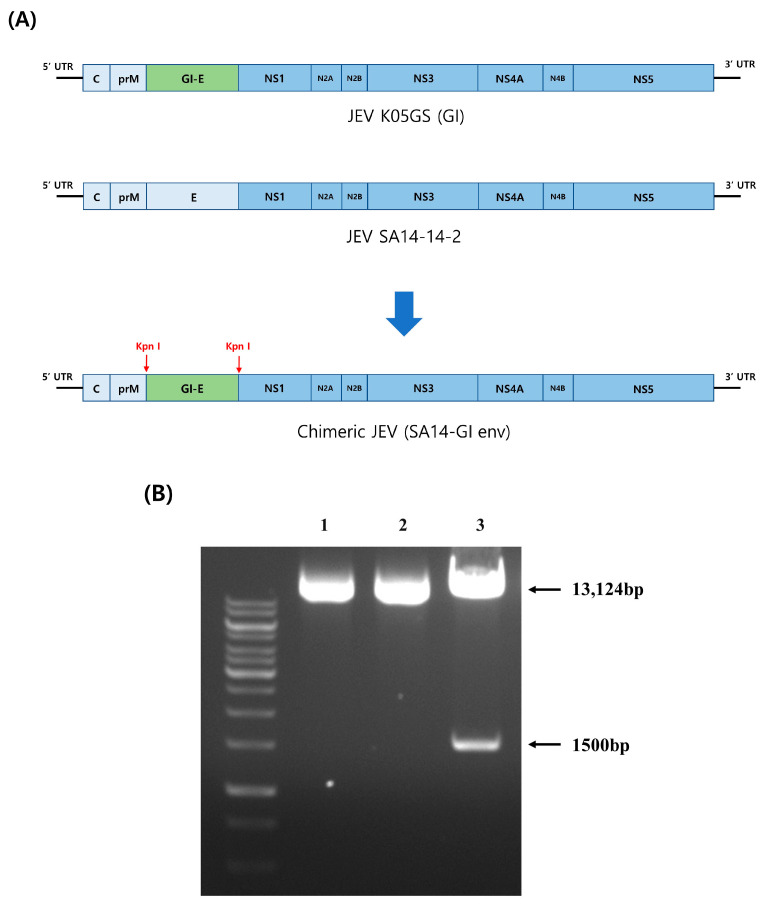
Schematics of two vectors utilized to form SA14-GI. (**A**) DNA encoding the envelope protein of JEV SA14-14-2 was removed and replaced with the codon-optimized DNA encoding the E region of the JEV GI strain (K05GS). (**B**) Agarose gel electrophoresis of plasmid DNA cleaved with a restriction enzyme. Restriction digestion of the SA14-GI env clone treated with enzyme (Kpn I). Lane 1: K05GS cDNA, Lane 2: SA14-14-2 cDNA, and Lane 3: fragment pattern of SA14-GI env plasmid.

To construct a cDNA clone, we obtained the gene encoding the full-length envelope protein of the JEV K05GS strain and a stable infectious cDNA clone of JEV SA14-14-2 after removing the E region with a polymerase chain reaction (PCR) (Figure 1A). To confirm the presence of the K05GS envelope protein in the pACYC184-JEV SA14-14-2 vector, we generated a clone (SA14-GI env cDNA) through transformation. Accordingly, the vector and insert proteins were proven to have equal sizes via enzyme (Kpn I) digestion (Figure 1).

### 3.2. Construction and Confirmation of Chimeric JEV

**Figure 2:**
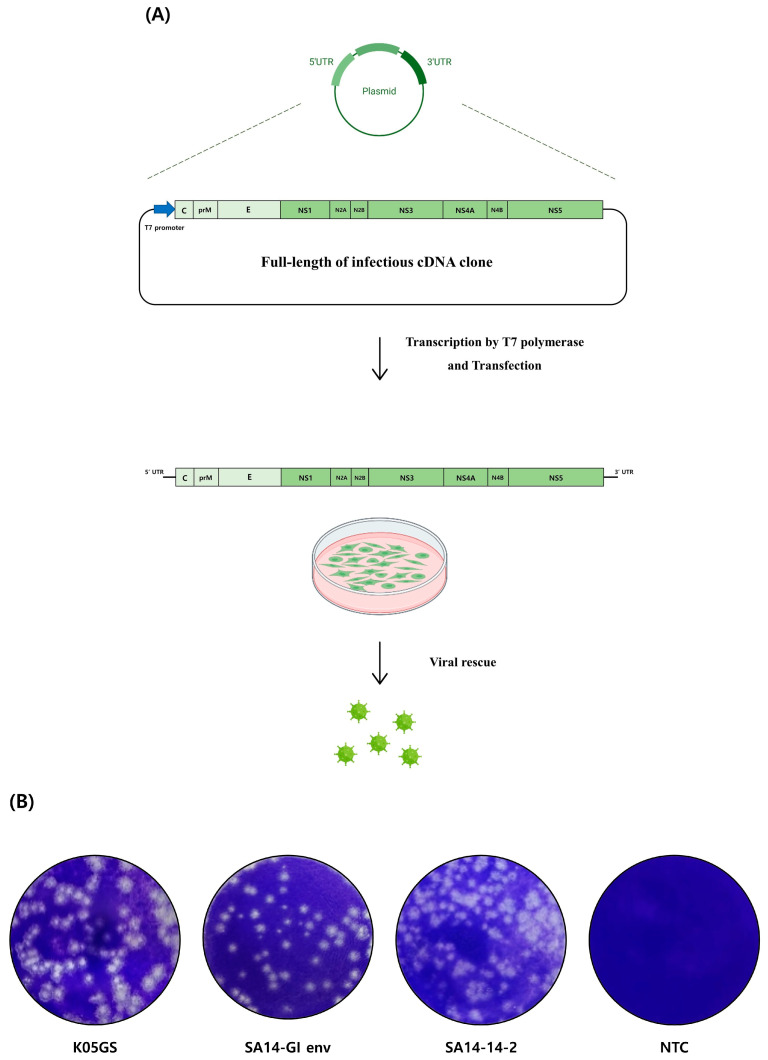
Expression of chimeric JEV in vitro. (**A**) Reverse genetic system establishment of reverse genetics for JEV. Infectious cDNA corresponding to the RNA genome of JEV was synthesized and engineered into a specific plasmid. Subsequently, in vitro transcription was utilized to artificially produce a full-length viral RNA transcript, which was then transfected into cells to produce infectious recombinant JEV virions. (**B**) Plaque phenotypes of K05GS, SA14-GI env, and SA14-14-2 strains in Vero cells.

The SA14-GI env plasmid encoding the full-length viral genome with the GI E protein was transfected into BHK-T7 cells to rescue the virus (Figure 2A). Rescued virions were cultured and harvested from Vero cells. To clarify the viral formation mechanism of SA14-GI env in vitro, we compared the plaques formed by infection using SA14-GI env with those formed by strains K05GS and SA14-14-2 in Vero cells. Plaques were formed by the SA14-GI env strain, which were similar to the plaques of the parental K05GS strain (Figure 2B), and the diameters of the plaques formed by the chimeric JEV strain were slightly smaller than those formed by the parental JEV strain.

### 3.3. Attenuation of Neurovirulence of the Chimeric JEV Strain in Mice

**Figure 3:**
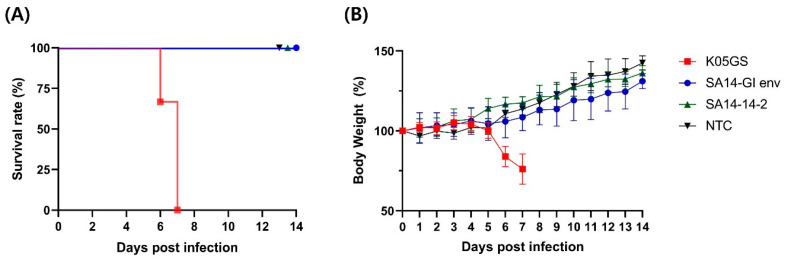
Murine neurovirulence of the chimeric JEV strain. (**A**) Survival rate of mice inoculated intracerebrally with 1 × 10^4^ PFU of K05GS (n = 6), SA14-GI env (n = 6), SA14-14-2 (n = 6), and negative control (n = 6). (**B**) Body weight of mice inoculated with JEV strains. Average change in body weight is presented as mean ± SD.

Evaluating whether engineered viruses are neurovirulent for vaccine development is important. Thus, we examined the neurovirulence of the chimeric JEV strains in vivo. Mice were inoculated with an equivalent dose of the K05GS, SA14-GI env, and SA14-14-2 strains via the intracerebral route and observed for 14 days. All six mice inoculated with the K05GS strain died within seven days of inoculation, whereas those inoculated with the SA14-14-2 strain or SA14-GI env strain survived. A significant loss in body weight was observed in K05GS-inoculated mice but not in SA14-GI env- and SA14-14-2-inoculated mice (Figure 3). These results indicated that the neurovirulence of the SA14-GI env strain was substantially reduced by the gene associated with the attenuated SA14-14-2 backbone.

### 3.4. Immunogenicity of SA14-GI Env Strain in BALB/C Mice

**Figure 4:**
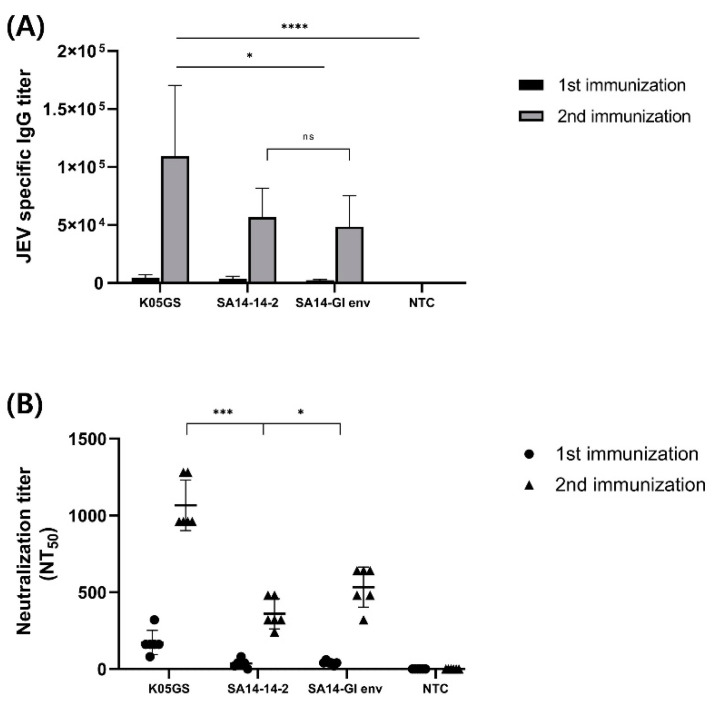
Immunogenicity of the SA14-GI env strain in vaccinated BALB/c mice. (**A**) JEV-specific IgG antibody responses in vaccinated mice detected using ELISA. BALB/c mice (6 mice/group) were immunized twice with K05GS, SA14-14-2, chimeric JEV, and normal BABL/c. (**B**) Neutralization activity of serum obtained from immunized mice against GI K05GS (* *p* < 0.05, *** *p* < 0.001, and **** *p* < 0.0001). NS: not statistically significant.

Six-week-old BALB/c mice were intraperitoneally immunized twice with the K05GS, SA14-14-2, or chimeric JEV strain. To investigate immunogenicity, we collected blood samples 7 days after each booster immunization, and the sera were provided for immunogenicity testing against JEV GI. Comparing the differences between the groups in the humoral immune responses by conducting ELISA analysis revealed that all groups’ JEV GI-specific IgG titers increased dramatically after each booster immunization. Moreover, compared with the parental strain, sera from the K05GS-inoculated group exhibited the highest IgG titer (*p* < 0.05, Tukey–Kramer post hoc test), and the immunogenicity of sera from the SA14-GI env-inoculated group was not significantly different from that of the SA14-14-2-inoculated group (Figure 4A). Similar results were observed in the neutralization analysis. Sera from mice inoculated with the K05GS strain had the highest NT50 against JEV GI (*p* < 0.001, Tukey–Kramer post hoc test), and the NT50 of sera from the SA14-GI env-inoculated mice was higher than that of the SA14-14-2-inoculated-group (*p* < 0.05, Tukey–Kramer post hoc test) (Figure 4B). These results revealed that the SA14-GI env strain induced higher levels of neutralizing antibodies against JEV GI in mice than the SA14-14-2 strain.

### 3.5. Protective Capacity of the GI K05GS Strain in SA14-GI Env-Vaccinated Mice

To analyze the protective efficacy of the chimeric virus against JEV GI, three groups of BALB/c mice were immunized twice at 2-week intervals i.p. with 1 × 10^5^ PFU of the K05GS, SA14-GI env, or SA14-14-2 strain. A PBS-inoculated group was adopted as a negative control. After 14 days of the last vaccination, immunized mice groups were challenged i.c. with 100LD_50_ of the K05GS strain. Hence, significant weight loss was observed in the PBS-inoculated mice group, exceeding 20.52% of their initial weight.

**Figure 5:**
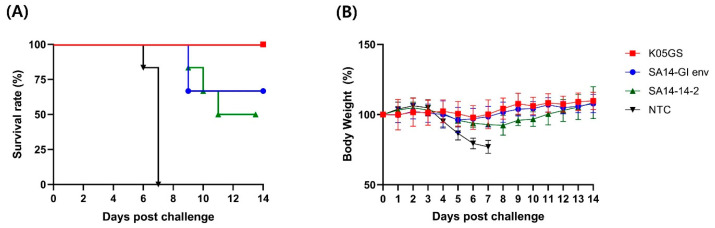
The GI K05GS strain challenge test in immunized mice. Immunized BALB/c mice were challenged with the K05GS strain and monitored for 14 days. (**A**) Survival rates of immunized mice after the challenge. (**B**) Body weight monitored daily for 14 days. Average change in body weight is presented as mean ± SD.

Furthermore, they developed immobility, scruffy fur, and a hunched posture, ultimately succumbing to death on Day 7. In contrast, all mice immunized with two doses of K05GS survived, whereas those in the SA14-14-2-inoculated group demonstrated a 50% survival rate, with three out of six deaths. Corresponding to previous results, the SA14-GI env group exhibited a higher survival rate, with two out of six deaths (Figure 5, Table 1). However, all mice in these groups demonstrated weight loss and clinical signs.

## 4. Discussion

JE is a zoonotic disease transmitted to humans and swine by mosquitoes [16,17,18]. The disease is prevalent in southern and eastern Asia and the western Pacific region, posing a considerable threat to national healthcare in these regions [19]. Moreover, it can cause viral encephalitis in humans and serious complications in reproduction systems in pigs [20]. The currently available JEV vaccines are based on the GIII strain. Inactivated mouse brain-derived vaccines have been utilized since 1954; they contributed to the successful containment of epidemic events and are still widely utilized today. However, it is being replaced by live or inactivated Vero cell-derived vaccines, due to high production costs, multiple dose requirements, and safety concerns, compared to other live vaccines. Recently, one research team developed a Vero cell-based recombinant live-attenuated JE vaccine that is effective against GV JEV infection [21].

The mosquito-isolated SA14-14-2 strain is widely utilized in the production of live-attenuated Japanese encephalitis vaccines and as a donor strain for replicating recombinant vaccines. The live-attenuated vaccine is a primary PHK cell-derived, attenuated live vaccine utilizing the SA14-14-2 strain, which was licensed in China in 1988 [22]. This vaccine is now licensed and available in more countries in Asia. The live recombinant chimeric vaccine is produced in Vero cells based on the yellow fever vaccine virus strain 17D (YF-17D), genetically modified to contain the prM/E proteins of SA14-14-2. It has been licensed and is increasingly utilized in Asian countries since its licensing in Australia in 2010 [22]. In China and Korea, JEV GI strains have become the dominant genotype, exceeding Type III strains [23,24,25]. Given the shift in genotypes, novel JEV GI strain vaccines must be developed to manage and contain this epidemic effectively.

Reverse genetics is a genetic manipulation technique, unlike conventional classical/forward genetics. The forward genetics system targets and modifies the gene sequence to obtain the phenotype and infer the function or regulatory mechanism of the gene [26]. It plays an important role in the development of vaccine platforms through genetic modification. It is being utilized in various fields to analyze and understand RNA viruses at the molecular level, such as research on the creation of new viruses and the mechanisms of viral replication and transmission. The advantage of vaccine development utilizing a reverse genetic system is that each gene segment can be easily cloned and stored after isolating the virus. Accordingly, samples can be stored and moved freely, and high-to-low-pathogenic virus conversion without modifying their antigenicity is possible by removing connecting peptides [26,27]. In addition, when a new modified virus appears, a vaccine can be prepared quickly with the point mutation method, based on the results of genetic analysis, without directly isolating the virus [14,26,27].

We constructed a chimeric JEV effective against JEV GI infections. Utilizing a reverse genetics system, we generated a recombinant GI/GIII intertypic SA14-GI env strain and encoded the E protein of a GI isolate, namely K05GS, utilizing the JEV SA14-14-2 vaccine strain. The chimeric viruses were validated in vitro and in vivo to evaluate their ability to serve as vaccines against GI JEV infections.

We confirmed that all unvaccinated mice inoculated with the strain (K05GS) died within seven days from the neurovirulence analysis. However, all the mice inoculated with SA14-GI env remained asymptomatic and survived until the end. This result indicated that the SA14-14-2 backbone extensively reduced the cytotoxicity of the parental virus in central neurons. Immunogenicity analysis indicated that the anti-JEV-specific IgG titer and NT50 against JEV GI were higher in samples collected from the K05GS-inoculated group than those observed in the SA14-14-2- and SA14-GI env-inoculated groups. No remarkable difference was observed in the IgG titer; however, the NT50 of SA14-GI env-inoculated mice against JEV GI was higher than that of SA14-14-2-inoculated mice, which could be attributed to the inclusion of a strain-specific epitope that recognizes neutralizing antibodies in the chimeric virus. In the challenge data, 100% survival was observed in the K05GS-inoculated group, while 66.6% of mice inoculated with 1 × 10^5^ PFU of the SA14-GI env strain and 50% of mice inoculated with the same dose of the SA14-14-2 strain survived. These results indicated that, compared with the existing vaccine strains, the SA14-GI env strain has a higher capability of blocking lethal GI K05GS infection in mice. Previous reports have suggested that the SA14-14-2 virus is a highly attenuated strain, where 24 amino acids have been substituted. These sites play an important role in the attenuation of neurovirulence of the SA14-14-2 strain [28,29,30]. A chimeric JEV with 16 amino acid substitutions in the untranslated regions (UTRs), capsid, and non-structural proteins that are included in the attenuated SA14-14-2 strain may have influenced the attenuated neurovirulence, as indicated in Figure 3 [31,32,33].

## 5. Conclusions

Here, we constructed the chimeric SA14-GI env for the first time and evaluated its protective and immunogenicity potential. The significant aspect of this study is that we developed a vaccine targeting the currently predominant JEV GI strain with a reverse genetics system. In conclusion, the SA14-GI env elicited higher humoral immunity and provided better protection than the current commercially available vaccine strain, SA14-14-2, indicating its potential as a new live-attenuated vaccine candidate. It could be a safer and more efficacious live vaccine candidate against JEV GI infection. Further animal studies can overcome the limitation of our study to assess the safety of the chimeric virus against JEV infection, including the evaluation of virus viremia (quantity and duration) before the clinical study. Further studies will be conducted to determine which of the 16 amino acid substitutions included in SA14-GI env are important factors for inducing attenuation.

## Figures and Tables

**Table 1:** Survival rates in immunized mice groups.

Group	Immunization	Challenge	Survival Rate (%)
Group 1	K05GS	K05GS	(6/6) 100
Group 2	SA14-GI env	K05GS	(4/6) 66.6
Group 3	SA14-14-2	K05GS	(3/6) 50
Group 4	PBS	K05GS	(0/6) 0

## Data Availability

Requests for data sharing should be addressed to the corresponding author.

## Supplementary Materials

The following supporting information can be downloaded at: https://www.mdpi.com/article/10.3390/vaccines11121827/s1, Table S1. Primers used for amplication of the envelope (E) protein and backbone of SA14-14-2.
