# Supplementary material for: Attenuated Chimeric GI/GIII Vaccine Candidate against Japanese Encephalitis Virus
Source: Vaccines (Basel). 2023 Dec 8;11(12):1827. doi: 10.3390/vaccines11121827 (PMC10747704; doi:10.3390/vaccines11121827)
Supplement: Supplementary file 1 [file vaccines-11-01827-s001.zip › vaccines-2694970-supplementary.pdf]

Table S1. Primers used for amplication of the envelope (E) protein and backbone of SA14-14-2

Primers	Sequences (5′-3′)	Tm (℃)	Product size (bp)
pACYC184_SA14-14-2_F	GGTACCACTGTAAGCCGGAGCGACCAACAGCAGGAGGATGG	65.1	13,122bp
pACYC184_ SA14-14-2_R	GGTACCGACACTGGATGTGCCATTGACATCACAAGAAAAGA	56.8	
Syn G1_Env_F	GCTTACAGTGGTACCTTTAACTGTCTGGGAATGGGCAACAGAG	71.4	1,500bp
Syn G1_Env_R	TCCAGTGTCGGTACCGGCATGCACATTGGTGGCCAGAAAC	76.4	
